# Blastic plasmacytoid dendritic cell neoplasm: a comprehensive review in pediatrics, adolescents, and young adults (AYA) and an update of novel therapies

**DOI:** 10.1038/s41375-023-01968-z

**Published:** 2023-07-14

**Authors:** Branko Cuglievan, Jeremy Connors, Jiasen He, Sajad Khazal, Sireesha Yedururi, Julia Dai, Sofia Garces, Andres E. Quesada, Michael Roth, Miriam Garcia, David McCall, Amber Gibson, Dristhi Ragoonanan, Demetrios Petropoulos, Priti Tewari, Cesar Nunez, Kris M. Mahadeo, Sarah K. Tasian, Adam J. Lamble, Anna Pawlowska, Danielle Hammond, Abhishek Maiti, Fadi G. Haddad, Jayatsu Senapati, Naval Daver, Naseema Gangat, Marina Konopleva, Soheil Meshinchi, Naveen Pemmaraju

**Affiliations:** 1 Division of Pediatrics, Department of Pediatric Patient Care, The University of Texas MD Anderson Cancer Center, Houston, TX USA; 2 Division of Pediatrics, Department of Pediatric Stem Cell Transplantation and Cellular Therapy, The University of Texas MD Anderson Cancer Center, Houston, TX USA; 3 Division of Radiology, Department of Abdominal Imaging, The University of Texas MD Anderson Cancer Center, Houston, TX USA; 4 Division of Internal Medicine, Department of Dermatology, The University of Texas MD Anderson Cancer Center, Houston, TX USA; 5 Division of Pathology, Department of Hematopathology, The University of Texas MD Anderson Cancer Center, Houston, TX USA; 6 Division of Pediatric Transplantation and Cellular Therapy, Department of Pediatrics, Duke University School of Medicine, Durham, NC USA; 7 Division of Oncology and Center for Childhood Cancer Research, Department of Pediatrics, Children’s Hospital of Philadelphia, Philadelphia, PA USA; 8 Division of Hematology/Oncology, Seattle Children’s Hospital, University of Washington, Seattle, WA USA; 9 Division of Pediatric Hematology/Oncology, and Hematopoietic Stem Cell Transplantation, City of Hope, Duarte, CA USA; 10 Division of Cancer Medicine, Department of Leukemia, The University of Texas MD Anderson Cancer Center, Houston, TX USA; 11 Department of Hematology, Mayo Clinic, Rochester, MN USA; 12 Department of Oncology, Montefiore Einstein Cancer Center, Bronx, NY USA; 13 Fred Hutchinson Cancer Research Center, Seattle, WA USA

**Keywords:** Leukaemia, Immunotherapy, Leukaemia, Cancer genetics, Targeted therapies

## Abstract

Blastic plasmacytoid dendritic cell neoplasm (BPDCN) is a rare hematologic malignancy that can involve the bone marrow, peripheral blood, skin, lymph nodes, and the central nervous system. Though more common in older adults, BPDCN has been reported across all age groups, including infants and children. The incidence of pediatric BPDCN is extremely low and little is known about the disease. Pediatric BPDCN is believed to be clinically less aggressive but often with more dissemination at presentation than adult cases. Unlike adults who almost always proceed to a hematopoietic stem cell transplantation in first complete remission if transplant-eligible, the majority of children can be cured with a high-risk acute lymphoblastic leukemia-like regimen. Hematopoietic stem cell transplantation is recommended for children with high-risk disease, the definition of which continues to evolve, or those in relapse and refractory settings where outcomes continue to be dismal. Novel agents used in other hematologic malignancies and CD123 targeted agents, including chimeric antigen receptor T-cells and monoclonal/bispecific antibodies, are being brought into research and practice. Our goal is to provide a comprehensive review of presentation, diagnosis, and treatment by review of pediatric cases reported for the last 20 years, and a review of novel targeted therapies and therapies under investigation for adult and pediatric patients.

## Introduction

Blastic plasmacytoid dendritic cell neoplasm (BPDCN) is a rare hematologic malignancy that typically involves the skin, peripheral blood (PB)/bone marrow (BM), lymph nodes, and central nervous system (CNS). Phenotypically different from other myeloid neoplasms, BPDCN arises from the inappropriate transformation of plasmacytoid dendritic cells (pDCs).

While the median diagnostic age of BPDCN is 65 years, it has been reported across all age groups, including infants and children. The incidence of BPDCN in pediatrics is extremely low—with fewer than 100 cases reported—and overall, little is known about the disease. Practitioners at pediatric institutions may be unfamiliar with BPDCN, and it may be mistaken for other aggressive hematological malignancies [1, 2]. No clinical, laboratory or imaging finding has been associated with prognosis. Given the rarity of this disease, there have been limited multi-center and collaborative group trials dedicated to this disease leading to a lack of a standardized treatment approach in both children and adults [3]. Additionally noting the limited number of cases, its inclusion in collaborative groups or multi-center studies seems unlikely at this time.

While it can be typical for trials to focus on either the youngest or oldest populations, there has been a growing trend to explicitly illustrate the outcome variations within the adolescent and young adult (AYA) population in numerous diseases. Many have demonstrated AYA patients to have superior outcomes relative to their older counterparts, especially when utilizing pediatric based regimens but, these patients often experience unique challenges, both psychosocially and secondary to treatment-related toxicities [4–9]. There are limited trials that direct focus specifically to the AYA population, with many outcome studies being retrospective or secondary analyses within existing larger population trials.

This review will cover the presentation of BPDCN in pediatric and AYA patients, including physical examination and imaging findings, morphologic and molecular pathologies, novel targeted therapies including their integration into existing traditional chemotherapeutic regimens, and discuss prior pediatric treatment strategies based on published reports over the last 20 years.

## Epidemiology

The World Health Organization established diagnostic criteria for BPDCN in 2008; since then, pediatric BPDCN has been more widely recognized and documented [10, 11]. BPDCN represents less than 1% of primary cutaneous skin lymphomas in adults; the exact incidence of BPDCN in children is unknown, owing to the frequently changing nomenclature and the lack of specific defining criteria before 2008, BPDCN may represent a larger number of the primary cutaneous lymphomas presenting in the pediatric age group [12–15]. Because cutaneous lesions are the first manifestation of the disease in more than 90% of patients, retrospective case reviews assessing the incidence of BPDCN may miss patients with extracutaneous disease [16].

BPDCN occurs in all age groups but is more likely to present in older adults; the median age at diagnosis is 65 years. Males tend to be affected slightly more than females, with some data showing an incidence ratio as high as 2.5:1. BPDCN does not appear to have any racial or geographic predisposition [16, 17]. Likewise, no environmental, inherited, or acquired genetic factors have been found to increase BPDCN risk. Retrospective review of European pediatric and adult patients with BPDCN on targeted therapy further confirms these demographic distributions [18].

BPDCN may not be an independent presentation; as many as 10–20% of patients with BPDCN have a previous diagnosis of acute myeloid leukemia (AML), chronic myeloid leukemia, chronic myelomonocytic leukemia (CML), or myelodysplastic syndromes [19–22], termed prior or concomitant hematologic malignancy [23, 24]. The interplay or transition between myeloid neoplasms and BPDCN, if any, has yet to be described. Other myeloid neoplasms, namely CML and *RUNX1*-mutant AML, have been known to show propagation of mature CD56-negative pDCs, underscoring the importance of differentiating these disease entities from BPDCN [16, 17].

**Fig. 1:**
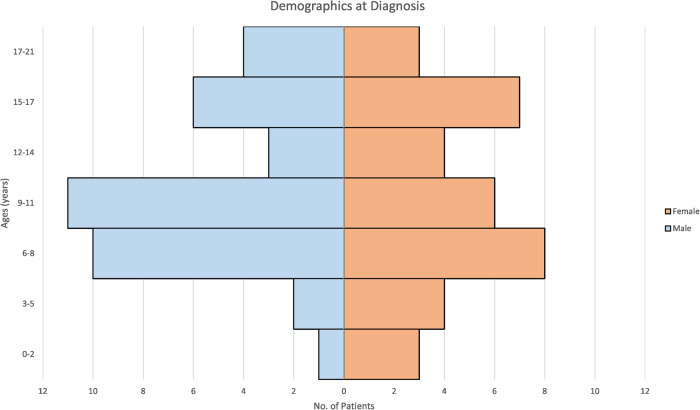
Pediatric BPDCN cases—age and sex distribution at diagnosis. The histogram depicts the distribution of age and sex at the diagnosis of pediatric cases of Blastic Plasmacytoid Dendritic Cell Neoplasm (BPDCN).

We identified 69 cases of BPDCN in patients aged 21 years or younger published in the last 20 years (Supplementary Table 1). These cases were evenly split between males (35 patients) and females (34 patients), which is different than that of the adult cohorts, as noted in Table 1. The mean and median ages at presentation were 10 years, a distribution of which can be seen in Fig. 1.

**Table 1:** Comparison of key characteristics in presentation and outcome between adult and pediatric patients with BPDCN.

Characteristic		Adult patients [123]	Pediatric and Adolescent Patients
Age (Years)	Median	66	10
	Range	19–86	0.6–18
Sex (M:F)		4:1	1:1
Presentation	Skin+ Only	35%	25%
	BM+	64%	75%
CNS+ During Therapy		33%	60%
Dead of Disease – Initial Therapy		66%	7%
HSCT at CR1		33%	20%
Overall Survival (Months)	Median	23	-
	Mean	-	78.8
	Range	0.9–158	1–132

Summary statistics of adult patients from Pemmaraju et al. (2022).

*BM* bone marrow disease, *CNS* central nervous system disease, *HSCT* hematopoietic stem cell transplant, *CR1* relapse from first successful therapy.

## Pathogenesis

BPDCN cells arise from myeloid lineages, specifically pDCs. Unlike conventional dendritic cells, pDCs can respond to viral infection with the aggressive production of interferon-α and -β [25], which seems to suggest that BPDCN stems from a viral trigger; however, no causative associations between infectious etiologies and BPDCN have been reported [26–28]. TCF-4, an E-box transcription factor, has an integral role in the proliferation of BPDCN [29, 30]. Similarly, the continuous expression of B-cell lymphoma 2 (BCL2) protein is vital to BPDCN, and this has given rise to the increased use of the BCL2 inhibitor venetoclax [31]. Other genotypic markers in BPDCN are fairly non-specific and thus do not offer actionable information for either elucidating the pathogenesis of the disease or improving its treatment [32].

## Pathologic features

### Skin and lymph nodes

**Fig. 2:**
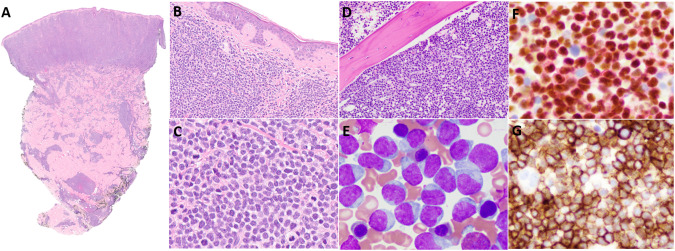
Histological Presentation Patterns of BPDCN. BPDCN involving the skin of a 14-year-old female. Staining with hematoxylin and eosin reveals a diffuse monotonous dermal-based infiltrate of medium-sized blastoid cells that spares the Grenz zone and epidermis and focally extends into the subcutaneous tissue (**A**, ×2; **B**, ×200). Occasional mitotic figures are present (**C**, ×500). Tumor cells are positive for CD4, CD56, and CD123 but negative for CD3, CD19, and myeloperoxidase (not shown). BPDCN involving the BM of a 16-year-old male. Staining with hematoxylin and eosin reveals an interstitial and diffuse infiltrate displacing hematopoietic elements (**D**, ×200;). **A** Wright-Giemsa–stained BM smear shows numerous blastic cells with irregular nuclear contours, immature chromatin, occasional small nucleoli, and variable light basophilic cytoplasm with occasional tail-like protrusions (**E**, ×1000). By immunohistochemistry, tumor cells are positive for TCF4/CD123 (**F**, ×500) and CD56 (**G**, ×500) but negative for CD3, CD19, and myeloperoxidase (not shown).

Histologically, cutaneous BPDCN lesions are characterized by a diffuse monotonous infiltrate of medium-sized blastoid cells that are centered in the superficial dermis, epidermis, and adnexal structures with a prominent Grenz zone. The infiltrate typically extends deep into the subcutaneous tissue [10, 33–35] (Fig. 2A, B). The neoplastic cells have scant cytoplasm and eccentrically positioned nuclei with ovoid or slightly irregular contours, fine chromatin, and one or more small nucleoli. [10, 34–36] Mitotic figures are variable, and necrosis can be present (Fig. 2C). In lymph nodes, BPDCN involves the medullary and interfollicular areas [34, 35].

### PB and BM

In the BM, BPDCN shows a diffuse and/or interstitial infiltration pattern, often replacing hematopoietic elements (Fig. 2D) [21, 34, 35]. Tumor cells may show a broad cytomorphological spectrum in Wright-Giemsa–stained smears; nevertheless, most cases consist of medium-sized blastoid cells with eccentric ovoid or irregular nuclei, open chromatin, and visible nucleoli. The cytoplasm is lightly basophilic and agranular, with occasional vacuoles and tail-like protrusions, often referred to as hand-mirror or tadpole morphology (Fig. 2E) [21, 34, 35].

### Immunophenotype

Immunophenotypically, BPDCN cells are positive for pDC-associated markers, including CD123, BDCA2 (CD303), and TCF4 [35]. CD123, the interleukin-3 (IL-3) receptor α-chain, is characteristically expressed at high levels [34, 37]. BPDCN cells are negative for myeloperoxidase (MPO), butyrate esterase, and naphthol-AS-D-chloroacetate esterase cytochemical stains [38]. CD303 (BDCA2), a pDC-specific lectin, is a useful and specific marker of BPDCN; however, its sensitivity may be limited owing to its frequent aberrant downregulation in BPDCN cells [34, 39, 40]. Recently, dual-color TCF4/CD123 immunohistochemical staining was proposed to be highly sensitive and specific for the detection of pDCs in formalin-fixed paraffin-embedded tissue sections (Fig. 2F) [29]. BPDCN cells also frequently express CD4, CD56 (Fig. 2G), HLA-DR, and TCL1. The neoplasm may also express various other lymphoid- or myeloid-related markers, including CD2, CD5, CD7, CD33, CD38, CD68, CD117, HLA-DR, and TdT. Nevertheless, by definition, BPDCN cells are negative for lineage-specific antigens for B cells (e.g., CD19), T cells (CD3), and myeloid cells (MPO) [35, 37]. BPDCN typically has a high Ki-67 proliferative index [35]. A few studies suggest that S100 protein expression is more common in pediatric BPDCN patients than in adult patients [10]. However, no statistically significant differences in the immunophenotype between the two age groups have been reported.

**Fig. 3:**
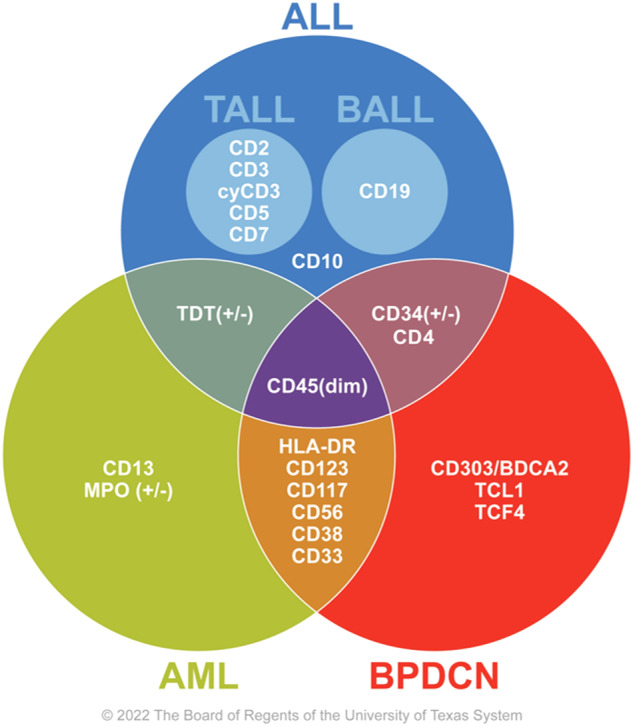
Typical cell surface markers of BPDCN and their overlap with those of other hematologic malignancies.

Immunophenotyping with multicolor flow cytometry is crucial in the initial diagnostic workup and for detecting minimal residual disease after therapy. The multi-antigen panels and gating strategies currently employed at MD Anderson for the detection of BPDCN were recently described in detail and include the markers CD4, CD33, CD45, CD56, CD64, CD123, CD303, and HLA-DR. In addition to CD56 expression, other aberrancies revealed by flow cytometry in most BPDCN cases include CD7 expression, decreased CD123 expression, decreased or absent CD38 expression, absent CD2 expression, and CD303 expression [37]. The overlap of the aforementioned markers among BPDCN and other hematologic malignancies is illustrated in Fig. 3.

### Genetic features

Because BPDCN rarely occurs in children, most of the information about the cytogenetic and molecular aberrancies in pediatric BPDCN is extrapolated from findings in adult cohorts. Most patients harbor chromosomal abnormalities detectable by conventional karyotyping, and up to 75% have a complex karyotype (defined as at least three chromosomal aberrations, including at least one structural aberration) [41]. In adult cohorts, the most frequently involved chromosomes are chromosomes 5, 6, 9, 12, 13, and 15 [41, 42]. Some of the most common findings are abnormalities affecting the 12p13 locus containing ETV6 [34, 41]. In one of the largest pediatric BPDCN studies evaluating 12 children with available cytogenetic data, 10 (83%) children had an abnormal karyotype, including six (50%) with a complex karyotype. Moreover, six children with an abnormal karyotype had abnormalities in chromosome 6 [10].

More recently, in a study that applied RNA sequencing–based comprehensive transcriptome analysis in 14 BPDCN patients (five children and nine adults), Suzuki et al. detected recurrent rearrangements involving MYB in all five children and four of the nine adults (44%) [43]. The resulting fusion genes in the pediatric cases were *MYB::PLEKHO1* (*n* = 3), *MYB::ZFAT* (*n* = 1), and *MYB::DCPS* (*n* = 1). Interestingly, except for one patient with t(1;6), who harbored a *MYB::PLEKHO1* fusion, metaphase analysis did not detect the translocations corresponding to these fusions. Further functional experiments in vitro showed that exogenous *MYB::PLEKHO1* expression in 293T cells led to the upregulation of several of MYB’s downstream targets, including NCAM1 (CD56), CD68, S1PR1 (CD363), and CXCR4 [43].

Next generation sequencing (NGS)-based mutation profiling of BPDCN in adults detected mutations involving multiple driver genes, including *TET2*, *ASXL1*, *NRAS*, and *ATM* [21, 44, 45]. Splicing factor mutations, such as *ZRSR2* have also been demonstrated to be common in BPDCN [46]. A few studies have analyzed pediatric cases using NGS. Suzuki et al. interrogated four pediatric patients using whole-exome sequencing and detected between six and 45 somatic mutations per patient. One patient had a *KMT2D* p.Cys1403Gly missense mutation, but no other driver mutations were identified [43]. More recently, in a whole-exome sequencing–based study of four pediatric patients, Liao et al. detected *KMT2A* mutations in all four patients and *IKZF2* mutations in two of the four patients [47]. Further investigation in larger pediatric cohorts is needed to elucidate the incidence and role of these mutations in pediatric BPDCN.

## Clinical presentation

The presentation of BPDCN is quite diverse and can involve the skin, BM, lymph nodes, and extra-nodal sites, including the CNS. Some pediatric patients have only BM involvement at diagnosis, but others can initially present with skin erythema and lymphadenopathy, which is often associated with fulminant disseminated intravascular coagulation (DIC), before rapidly developing BM and CNS disease. These presentations may vary within the pediatric age group, but this is difficult to define given the limited number of cases. In contrast to adult patients, pediatric patients often present with multiorgan involvement, DIC, and tumor lysis syndrome as demonstrated in our case review.

### Cutaneous involvement

About 75–90% of BPDCN patients in all ages have cutaneous involvement at presentation [16, 25]. Cutaneous involvement typically includes solitary, localized, or generalized vascular plaques or tumors that commonly occur on the head and neck or upper trunk. Many of these lesions demonstrate a characteristic bruise-like violaceous hue owing to intratumoral hemorrhage [48]. Although skin lesions can take longer to clear than disease noted in the PB, BM, and lymph nodes, these lesions can be used as an additional marker of treatment response [49]. In previous reviews, pediatric BPDCN patients without cutaneous lesions had better clinical outcomes compared to those with cutaneous involvement. In contrast, survival outcomes did not differ significantly between adults with and without cutaneous involvement [49, 50]. In our review of the reported pediatric cases, 52 cases (80%) noted positive skin findings, in which the vast majority of cases explicitly documented skin exam at diagnosis. As compared to the literature in adult patients, this pediatric population had a slightly lower rate of skin-only presentations (25% vs 35% in pediatric and adult patients respectively, as noted in Table 1).

### Hematolymphoid

Many BPDCN patients have cytopenias, reactive lymphadenopathy, and/or splenomegaly, and about half present with disease identified in lymph nodes [51]. Hepatic involvement is more common in patients with extensive BM disease. These patients can present with significant coagulopathy and can develop life-threatening DIC [52]. In our review of the available pediatric case reports, 43 (75.4%) and 15 (39.4%) cases had documented disease in BM and PB respectively. It is again important to note that only 57 (82.6%) and 38 (55%) of cases reported BM and PB at diagnosis, perhaps the latter being related to the belief that if the bone marrow is positive, the entire hematologic compartment is positive. This is slightly higher than presented adult data as noted in Table 1.

### CNS involvement

Reported rates of CNS involvement range from 20% to 60%; the actual rate is unknown, as lumbar puncture at diagnosis has not historically been part of a standard of care workup [53–56]. Rarely, patients can have clinical features, such as headaches, confusion, seizures, syncope, intracranial hypertension, and vision problems, which can be associated with ocular and/or imaging findings that may additionally alert providers to CNS disease [53–58]. The prevailing approach to assessing the prevalence of CNS disease relies primarily on the reporting of cases in which patients have undergone specific assessments targeting the central nervous system. While this method may not capture the complete picture, it remains the primary available avenue for prevalence estimation.

In a retrospective case review of adults, cerebrospinal fluid (CSF) analysis was performed in 29 patients, resulting in 13 patients diagnosed with CNS disease at frontline therapy and 10 patients diagnosed during treatment. This suggests an approximate prevalence of 34–45% for CNS disease in this cohort. The remaining and larger portion of the cohort was not assessed for CNS disease, leading to an unknown CNS status. It is possible that the rate of CNS involvement is even higher in cases of relapsed disease [54]. Interestingly, in one review of adult patients with BPDCN, 100% of those patients experiencing a relapse were studied and found to have confirmed CNS disease, all by flow cytometric analysis of CSF [55].

**Fig. 4:**
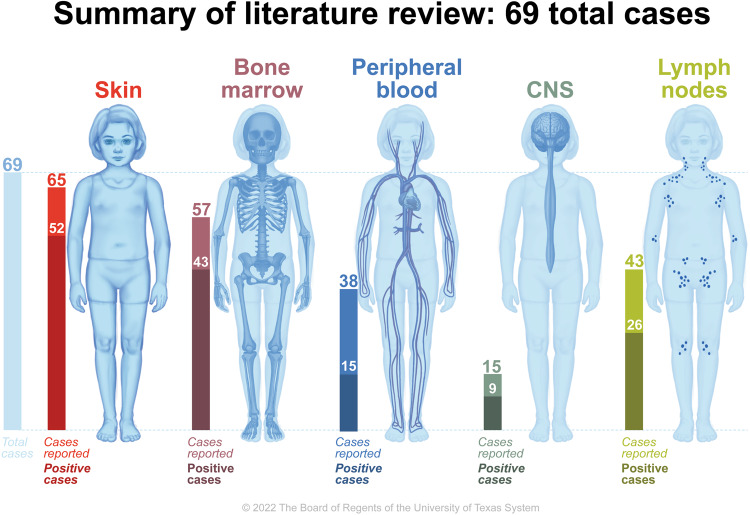
Locations of BPDCN involvement at diagnosis in a review of 69 pediatric cases.

In the review of pediatric case reports, 15 patients were assessed for CNS disease. Nine of them demonstrated CNS involvement, indicating an estimated prevalence of ~60% for CNS disease within the pediatric subset. Of the pediatric patients with CNS involvement, the vast majority were asymptomatic and diagnosed through CSF cytology. In those pediatric patients without CNS disease at presentation who experienced a medullary relapse, none had new CNS findings. In those with known CNS disease, three patients experienced relapse, and only one was noted to have to have continued CNS positive disease as the other two did not receive any further therapy or CNS evaluation. The high incidence of positive findings in reported cases underscores the importance of conducting CSF cytology and imaging (if appropriate) at diagnosis, during therapy, and in cases of relapse. These measures are necessary to accurately determine the incidence and behavior of CNS disease in the pediatric population. This is summarized in Fig. 4.

### Other Findings

**Fig. 5:**
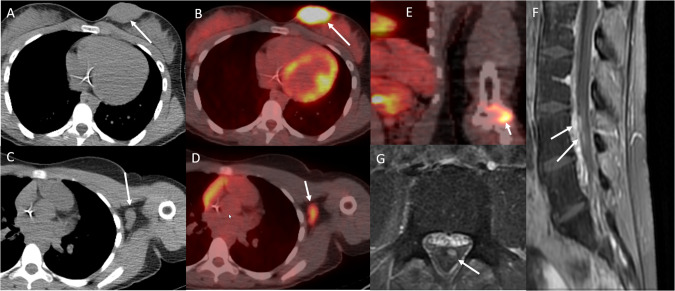
Radiologic Manifestations of BPDCN. Axial CT and fused PET/CT of a teenage female with BPDCN show nodular skin and subcutaneous soft tissue thickening in the left breast that is fluorodeoxyglucose-avid (arrows in **A** and **B**) and associated with fluorodeoxyglucose-avid left axillary lymphadenopathy (arrows in **C** and **D**) as well as a fluorodeoxyglucose-avid bone lesion in the left distal humerus (arrow in **E**). MRI of the spine shows enhancing nerve roots in the lumbar spinal canal, consistent with leptomeningeal disease (arrows in **F** and **G**).

**Fig. 6:**
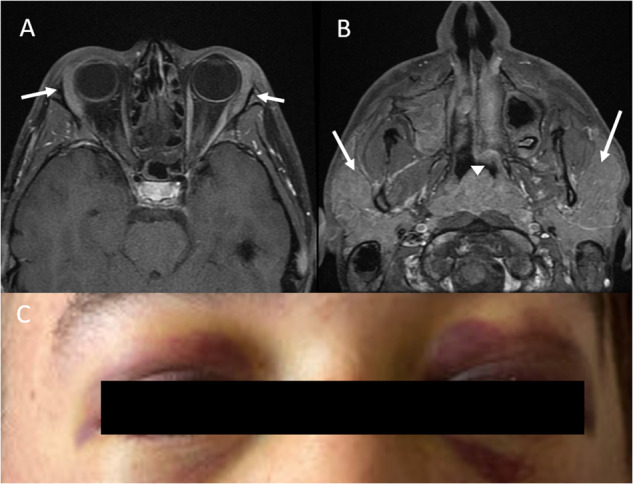
Imaging Findings in CNS BPDCN. Axial MRI of the face of a teenage male with BPDCN shows diffusely enlarged lacrimal glands (arrows in **A**), diffusely enlarged parotid glands (arrows in **B**), and a diffusely enlarged adenoid (arrowhead in **B**). CT of the neck, chest, abdomen, and pelvis reveals multicompartmental lymphadenopathy (involving the cervical, axillary, hilar, mediastinal, periportal, retroperitoneal, and iliac chain and inguinal lymph nodes) and hepatosplenomegaly (not shown). Brain and spine MRI reveal no leptomeningeal disease. Periorbital ecchymosis (“raccoon eyes”) at presentation in a patient later diagnosed with BPDCN (**C**).

BPDCN can infiltrate a wide range of extramedullary tissues and organs, including the orbital area, lacrimal ducts, nasopharynx, lungs, uterus, and ovaries [59]. Patients can present with vision loss and periorbital ecchymosis, or “raccoon eyes” (Figs. 5 and 6) [60, 61].

## Pathology

BPDCN can be challenging to diagnose, especially in patients who have atypical or nonspecific skin lesions. Thus, cytologic evaluation and immunophenotyping are required to obtain a definitive diagnosis. Unlike in other hematologic neoplasms, the various markers that comprise the immunophenotypic fingerprint of BPDCN cannot be used to diagnose the disease, as these markers are present on normally functioning cells [17]. Cytologic evaluation is challenging, as the morphology of BPDCN may mirror that of other cell types. As our ability to summarize the genetic expression profiles of various diseases improves, more accurate diagnoses may be possible. While it appears to vary among patients, a prototype phenotypic presentation includes cells that are negative for CD3, CD13, CD16, CD20, lysozyme, and MPO but positive for CD4, CD56, CD123, CD303, and TCL1A [10, 17, 62–64].

The molecular and genetic phenotypes of BPDCN can overlap with those of acute leukemia (including myeloid, lymphoid, and mixed phenotype leukemia) and cutaneous myeloid sarcoma (leukemia cutis). Patients may present with PB showing myeloid appearing blasts (±CD33, CD56, and CD123), T-cell and NK-cell leukemia/lymphoma (+CD2 and CD56). These are also often associated with infectious etiologies, including Epstein-Barr virus causing pDC proliferation. This overlapping phenotype can be present with lymph node, BM, cutaneous infiltration (typically negative for CD56 and less blastic in appearance), chronic myeloid leukemias, blastic mantle cell, and high-grade lymphomas [17, 63, 65].

## Imaging

For both pediatric and adult BPDCN, reported imaging findings vary and are limited to isolated case reports [48, 54, 66]. These findings are similar to those described for leukemia and lymphoma. In patients with skin and/or subcutaneous soft tissue involvement, computed tomography (CT) reveals nodular or plaque-like skin thickening, which is fluorodeoxyglucose-avid on positron emission tomography (PET)/CT, as seen in Fig. 5 [67–69]. As with other lymphomas and leukemias, the extent of multifocal lymphadenopathy and BM involvement can be assessed with CT, PET/CT, or magnetic resonance imaging (MRI) as seen in Fig. 6 [67, 69]. Imaging can also confirm the presence or absence of hepatomegaly and/or splenomegaly. MRI may or may not show anatomic abnormalities corresponding to clinical manifestations of CNS involvement. Reported MRI findings include leptomeningeal enhancement [70], optic nerve and orbital involvement [60], areas of restricted diffusion in the middle cerebral artery territory [71], and no pertinent findings [72]. The reported forms of lung involvement have included both diffuse ground glass or interstitial lung opacities and well-defined nodules [73, 74]. Other reported sites of disease have included the nasal cavity and paranasal sinuses, nasopharynx, testis, and multifocal visceral involvement [75–77]. The imaging findings for two pediatric patients with BPDCN are shown in Figs. 5 and 6. In our review of the pediatric case presentations, 26 (60.5%) had positive lymph node disease, noted either through imaging and/or direct tissue biopsy; these findings were documented in 43 (62.3%) of case reports.

## Treatment

### Systemic therapy

Given the small numbers of pediatric patients with BPDCN and the lack of randomized clinical trials or even robust case series, the regimens used to treat pediatrics patients with BPDCN vary dramatically by practitioner and center. Regimens are often based on the disease’s response to previous treatment, as illustrated in Supplementary Table 1. Much of the literature on BPDCN in adults discusses utilizing acute leukemia or lymphoma-based regimens [78]. Chemotherapy regimens used to treat AML, adult acute lymphoblastic leukemia (ALL) (e.g., hyper-CVAD [hyperfractionated cyclophosphamide, vincristine, doxorubicin, and dexamethasone]), and non-Hodgkin lymphoma (NHL) have been used in the treatment of BPDCN [78]. Given the aggressive nature and poor outcomes of BPDCN in adults, these patients typically receive chemotherapy followed by consolidation with hematopoietic stem cell transplantation (HSCT) [26, 63].

In pediatric patients with BPDCN, high-risk ALL-based therapies with multi-drug combinations and maintenance, including CNS prophylaxis, have yielded encouraging survival outcomes [10, 26, 79]. For example, St. Jude’s Children’s Research Hospital documented four patients, age 6–11 years, who were treated with an in-house ALL-like protocol. All patients had disease remission. Three had complete remission (CR) for 4.2 to 11.1 years, and the fourth was lost to follow-up [80]. Similarly, in a study of 29 pediatric patients with BPDCN by Jegalian et al., 12 of 14 patients who received ALL-like regimens achieved a CR [10, 81, 82]. After a follow-up ranging from 9 months to 13 years, only one patient with multiorgan disease died. For all 14 patients, the event-free survival (EFS) and overall survival (OS) rates were 64% and 72%, respectively. It is important to note that per this case collection, children who received AML-like regimens had increased rates of adverse outcomes, notable for poor/no response or short interval relapse [83–85].

### Hematopoietic stem cell transplant

The role of HSCT and its optimal timing in pediatric patients with BPDCN are unclear. In their retrospective study of 29 pediatric BPDCN patients, Jegalian et al. found that of six patients who underwent consolidation with allogeneic HSCT after remission was achieved, four had EFS of at least 5 years. The OS in the HSCT group was 67% versus 74% in the non-HSCT group, but this is confounded by disease presentation, initial therapy selected, and timing of HSCT. The authors recommended that allogeneic HSCT should be reserved for patients in second CR, those who do not achieve CR, or whose disease responds slowly to upfront ALL-based therapy [10]. In a systematic literature review, the median survival duration of adult and pediatric patient with BPDCN who underwent allogeneic HSCT (37.8 months) was significantly longer than that of those who did not undergo HSCT (21.1 months; *p* ≤ 0.01) [66]. More recently, investigators at MD Anderson Cancer Center assessed the outcomes of 17 adult BPDCN patients who underwent allogeneic HSCT and reported that the 5-year progression-free survival and OS rates were both 80% for patients who received HSCT after having a first CR but both 0% for patients who did not receive HSCT after having a first CR [86]. Given that pediatric patients with BPDCN have better overall outcomes than adult patients with BPDCN, allogeneic HSCT after a first CR should be reserved for pediatric patients with BPDCN who have high-risk features, the definition of which has historically been practitioner dependent.

There appears to be a group of pediatric patients with BPDCN who have an increased risk of progression, multiorgan involvement, minimal residual disease positivity, and/or refractory disease whose outcomes are universally worse [87]. As such, work is needed to investigate the ability to prognosticate outcomes. In these patients, induction of a molecular remission prior to consolidative therapy with HSCT should be considered. In this setting, weekly induction therapy with a four-drug ALL-based regimen is often not sufficient to quickly reduce tumor burden, control disease progression, and stop DIC. Treatment with NHL-based regimens or hyper-CVAD, which comprise a combination of consecutive cytotoxic chemotherapies given in combination with novel agents and followed by HSCT, have yielded favorable results. For example, in a 2010 NCI study, four of six patients who initially received NHL-based regimens, eventually achieved a CR. Within this cohort, three underwent HSCT (one after a first CR to frontline therapy, one after a first CR after transition to ALL-based therapy, and one after a second CR after relapse and eventually restarting the regimen) [88–91].

Given the increase in novel agents and clinical trials, more targeted salvage therapy (e.g., tagraxofusp, venetoclax) may be considered before allogeneic HSCT, especially in patients who previously received ALL-based regimens.

### Management of CNS disease and relapse

CNS prophylaxis, especially as employed by ALL-based regimens, has shown to play a key role in BPDCN treatment since the disease has a proclivity to invade the CNS. Recently, multiple experts have recommended that the standard treatment for BPDCN include routine a screening lumbar puncture and prophylactic intrathecal chemotherapy, as these measures have implications for survival. Given the heavy use of acute leukemia based regimens as primary therapy and their superior outcomes, many of which include CNS prophylaxis, the actual rate of CNS disease may be underrepresented in this literature [53–55]. As with acute leukemia, all newly diagnosed BPDCN patients should undergo CNS evaluation with CSF analysis with consideration of flow cytometry, since CNS involvement at diagnosis can be largely asymptomatic. In addition, mirroring AML and ALL-based regimens, CNS prophylaxis should be employed with intrathecal chemotherapy [83, 92, 93]. In addition, with the advent of novel therapeutics, further evaluation will be needed regarding these agents’ ability to penetrate the CNS and thus aid in prevention and treatment of CNS disease. Specifically, consideration of their ability to cross the blood-brain barrier and the success to which they can function in this setting will guide recommendations of further CNS treatment and prophylaxis in any salvage regimens.

### Review of treatment in pediatric published cases

**Fig. 7:**
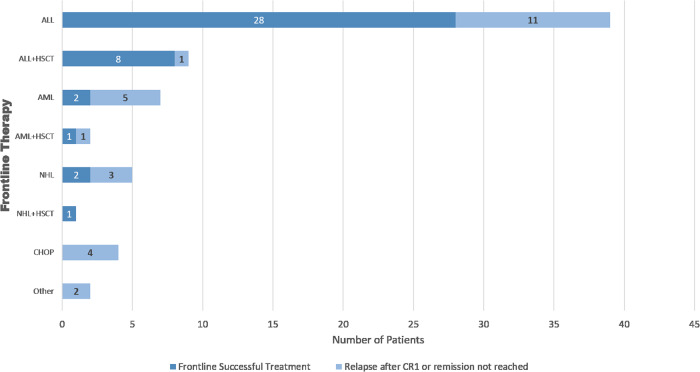
Outcomes from initial type of therapy regimen utilized.

Among the 69 pediatric patients with BPDCN we identified in our literature review (Supplementary Table 1), 48 (70%) received ALL-based regimens as their initial treatment, and of those patients, 44 (91%) achieved a first CR during this treatment. Nine patients (13%) received AML-based regimens, and the remaining patients received NHL-based regimens, CHOP (cyclophosphamide, doxorubicin hydrochloride, vincristine sulfate, and prednisone), or other regimens. Overall, 57 patients (83%) achieved a CR during the first regimen, and of these patients, 42 (61%) were relapse-free at the time of publication, including 10 that underwent HSCT. Six patients (9%) had partial responses, and one patient had no response. Of the patients who achieved a first CR, 14 (25%) experienced relapse. Outcomes of the first therapy utilized is noted in Fig. 7.

In those that had documented CNS disease at diagnosis, six received ALL-based regimens reaching complete remission (CR) without relapse, one of which received a hematopoietic stem cell transplant (HSCT) at CR1 coupled with 12 months of CNS prophylaxis. Two received ALL-based regimens with subsequent relapse at 5 and 15 months without salvage therapy pursued, and one received AML-based therapy experiencing multiple, CNS positive relapses, ultimately dying of disease at 36 months after receiving three salvage regimens and consolidating HSCTs.

Salvage therapy varied, but many patients received ALL-based regimens. Four patients (6%) died from disease during the primary treatment, and another 13 (19%) died during subsequent treatment after experiencing an event after primary treatment. Among the 21 patients who underwent HSCT, 14 (66.6%) underwent HSCT after CR1, and 7 (33.3%) underwent HSCT after CR2. Two underwent a second transplant after experiencing relapse, both having initially received their transplant after achieving CR1.

## Emerging agents

### Tagraxofusp

Unlike normal mature myeloid cells and macrophages, BPDCN is characterized by the high expression of CD123 (IL3RA) [94]. Tagraxofusp is a fusion protein consisting of a truncated diphtheria toxin and recombinant human IL-3 [95]. After tagraxofusp binds to CD123, the cell initiates receptor-mediated endocytosis. Because myeloid leukemia progenitors overexpress IL-3R, tagraxofusp was initially evaluated in patients with AML and myelodysplastic syndromes and found to be well tolerated [95]. Preclinical studies showed tagraxofusp to have antitumor activities in BPDCN cells in vitro and in vivo [96]. The most commonly reported adverse events are elevated liver enzymes, hypoalbuminemia, peripheral edema, and thrombocytopenia. Its most critical side effect is capillary leak syndrome, which can be fatal [97].

In one pilot phase 1 study published in 2013 [98], three of four heavily pretreated BPDCN patients achieved a CR lasting 1–5 months. In another prospective study of 11 patients with BPDCN, 7 achieved a CR and two had partial responses, following a single course of tagraxofusp, and the median response duration was 5 months [99]. Given these promising results, Pemmaraju et al. conducted an open-label, multi-cohort clinical trial of multiple cycles of tagraxofusp in 47 patients with untreated or relapsed BPDCN [97]. Tagraxofusp was given until disease progression or unacceptable toxic effects, and 32 of 37 patients received it as first-line treatment. The overall response rates for the patients with untreated BPDCN and those with relapsed BPDCN were 90% and 67%, respectively. Notably, 45% of the untreated patients who had a response later underwent HSCT. On the basis of this study, tagraxofusp was approved by the U.S. Food and Drug Administration for the treatment of BDPCN in adults and in children age 2 years or older.

Owing to the scarcity of the disease, there is little experience with tagraxofusp in pediatric patients with BPCDN. Sun et al. reported on the use of tagraxofusp in pediatric patients in three cases [100]. All patients received infusions of tagraxofusp (12 μg/kg/day for 5 days every 2–3 weeks). One achieved a CR and underwent HSCT, another had a transient response, and one had no response, the last of which had notably relapsed and refractory disease at treatment onset. Adverse events were mild and could be controlled with premedication. Pemmaraju et al. reported a multicenter, retrospective case series that included six pediatric patients age 10–21 years [101]. Of these patients, one achieved CR, two had stable disease, and three did not have a response. Three of the patients were bridged to HSCT, and five were alive at the time of the report. This limited evidence suggests that although tagraxofusp is well tolerated and effective in the pediatric population, its effects are transient, necessitating bridge therapy and HSCT. An ongoing open-label phase 1 trial (NCT05476770) is investigating tagraxofusp alone and in combination with other agents in pediatric patients with CD123-expression hematologic malignancies, including BPDCN.

### Venetoclax and azacitidine

A gene expression analysis of BPDCN revealed the upregulation of cyclin D1, a master regulator of cell cycle progression and BCL2 [102]. BCL2 family proteins regulate the intrinsic mitochondrial apoptotic response [103]. One study showed that primary BPDCN cells depend on BCL2 and are sensitive to BCL2 inhibition both in vitro and in vivo [31]. Theoretically, the hypomethylating agent azacitidine increases leukemia cells’ dependence on BCL2 by synergistically inhibiting the pro-survival proteins myeloid cell leukemia 1 and BCL-XL [104].

Venetoclax, a selective small-molecule inhibitor of BCL2, is approved by the U.S. Food and Drug Administration for the treatment of chronic lymphocytic leukemia and untreated AML [105, 106]. Venetoclax is generally well-tolerated, even in older, heavily pretreated patients. Key adverse events include nausea, thrombocytopenia, neutropenia, and febrile neutropenia [105]. Given its good safety profile, venetoclax can be used in patients who are not eligible for tagraxofusp and HSCT. A few case reports have described the successful use of venetoclax in BPDCN patients [31, 107–110]. Pemmaraju et al. reported their experience with venetoclax and azacitidine in 10 patients (median age, 70 years) whose baseline co-morbidities, including renal failure, cardiac disease, and hypoalbuminemia, made them ineligible for tagraxofusp [111]. The combination of venetoclax and azacitidine was able to bridge three patients to allogenic HSCT. An ongoing single-arm phase 1 trial (NCT03485547) is investigating venetoclax in combination with other agents [112, 113].

Venetoclax has also been used in pediatric patients with BPDCN. Alba et al. reported one 11-year-old male with BPDCN who initially received an ALL-based regimen followed by allogeneic HSCT but then had relapse in his testes. Because tagraxofusp was unavailable, he achieved a CR with hyper-CVAD in combination with venetoclax for 3 cycles and then underwent a second allogeneic HSCT [78].

### Tagraxofusp plus venetoclax and azacitidine

Togami et al. found that tagraxofusp resistance in BPDCN was regulated by the DNA methylation and downregulation of diphthamide genes rather than the loss of CD123 [114]. They also showed that this resistance pattern can be reversed by azacitidine in xenograft models.

On the basis of these findings, Lane et al. conducted a phase 1b trial of tagraxofusp combined with azacitidine or with azacitidine and venetoclax in patients with myeloid neoplasms, including BPDCN, of which 33 patients were enrolled [115]. The main adverse events were cytopenia, elevated liver enzymes, and capillary leak syndrome. Among the 11 (33%) patients who experienced capillary leak syndrome, eight (24%) had grade 2, two (6%) had grade 3, and one (3%) had grade 4. Among three patients with relapsed or refractory BPDCN who received tagraxofusp plus azacitidine and venetoclax, two had responses and proceeded to allogenic HSCT. Although tumor lysis syndrome is still a major concern with tagraxofusp plus azacitidine and venetoclax, this trial proved that with close monitoring and the early implementation of supportive care, the combination is feasible and has encouraging results.

### Post-HSCT maintenance with tagraxofusp

Because BPDCN is such an aggressive disease, the current treatment strategy is to start with a high-risk ALL-based regimen. In pediatric patients with high-risk features and/or refractory disease, subsequent therapy would involve bridging therapy with tagraxofusp plus azacitidine and venetoclax, and referral for allogeneic HSCT after the first CR. Although HSCT prolongs remission, relapse after HSCT still happens, especially in patients who had not reached remission prior to undergoing transplant. An ongoing phase 2 trial (NCT04317781) is investigating the effects of tagraxofusp after allogeneic HSCT in patients with BPDCN [116].

### IMGN632

Because BPDCN overexpresses CD123, researchers are investigating another agent targeting CD123: the antibody-drug conjugate IMGN632. IMGN632, a conjugate of a CD123-binding antibody and a novel DNA-alkylating payload, is effective in xenograft models of ALL and AML [117, 118]. Pemmaraju et al. studied the use of IMG632 in 23 patients with relapsed or refractory BPDCN (median age, 73 years), of whom 43% previously received tagraxofusp [119]. It is important to note that due to different drug structures and mechanisms of targeting, failure of tagraxofusp does not negate the ability to utilize this new agent. The most common adverse events were nausea, peripheral edema, and infusion-related reactions; no grade 3 or higher treatment-related adverse events and no capillary leak syndrome were observed. In this heavily pretreated group of patients with relapsed or refractory BPDCN, seven patients had objective responses lasting 3–9 months without HSCT. Three of the ten patients who previously received tagraxofusp had responses. Given its reassuring safety profile, IMGN632 may be a good option for heavily pretreated patients with relapsed or refractory BPDCN.

### Chimeric antigen receptor T-cell therapy

CD123-directed chimeric antigen receptor (CAR) T cells have been tested in adults with AML in several trials, some of which also included patients with BPDCN. These trials investigated different CARs with different safety switches, including CD20, EGFRt, and ROR8 [120]. Significant side effects included cytokine release syndrome. In a phase 1 trial (NCT03203369), one patient with BPDCN who received allogeneic T-cells with a second-generation CAR (CD123 scFv-41BB-CD3 ζ) developed cytokine release syndrome and died 9 days after cell infusion [121]. However, some CAR T-cell products have shown antitumor effects in patients with BPDCN. For example, two patients who received MB-102 (NCT04109482), a CD123-directed, autologous CAR T-cell therapy, only experienced reversible toxicities (e.g., less than or equal to grade 3), and achieved CR [120].

CD123-directed CAR T cells are also being investigated in pediatric patients with BPDCN. In the CATCHAML (CD123-Directed Autologous T-Cell Therapy for Acute Myelogenous Leukemia) trial (NCT04318678), researchers are evaluating the safety and tolerability of autologous CD123-directed CAR T cells in patients younger than 21 years with CD123-expressing hematologic malignancies, including BPDCN. They are using a second-generation CAR with a CD28 H/TM region and a CD28 ζ signaling domain to treat recurrent or refractory CD123-positive disease [122]. In another trial at the Children’s Hospital of Philadelphia, researchers are evaluating CD123 CAR-4.1BB-CD3z in pediatric patients with AML. Most of the trials in pediatric patients are still recruiting, and their results have not been published [120]. While this trial currently excludes patients with BPDCN, any documented efficacy may lead to further study of this modality in pediatric and AYA patients with BPDCN.

### Emerging drug targets

Other promising new therapeutic targets in BPDCN include CD38, HA-1H, CD56, and ILT3, but little has been reported about their potential use in the pediatric population [120].

## Conclusions

Much of the ongoing research in the treatment of pediatric BPDCN stems from advances in identifying therapeutic targets, developing novel targeted agents, overcoming the unique diagnostic challenges of the disease, and improving our knowledge of disease outcomes with therapeutic approaches. For pediatric patients with BPDCN, the best chance for CR still appears to be a high-risk ALL-based regimen followed by a period of observation, especially given these patients’ high rate of CNS involvement at diagnosis. Determining prognosis at time of diagnosis remains difficult, and the factors that predict response to therapy and overall prognosis remain unclear. The list of therapeutic options is growing for patients who do not achieve CR or who have relapsed or refractory disease that (at least initially) precludes HSCT. Areas that remain in high need of evaluation include the role and timing of HSCT with targeted therapies, the role of these targeted therapies in the frontline setting, and the impact these therapies have on site-specific disease presentations, including CNS disease. Recently the North American Blastic Plasmacytoid Dendritic Cell Neoplasm Consortium, a multi-disciplinary group of disease experts, was formed to define standard of care as well as identify these critical research questions [2]. We hope their inclusion of pediatric and AYA specialists will foster further development of clinical trials, novel therapies, and focus on BPDCN in the younger age group.

## Supplementary information

Supplementary Table 1
