# Supplementary material for: Blastic plasmacytoid dendritic cell neoplasm: a comprehensive review in pediatrics, adolescents, and young adults (AYA) and an update of novel therapies
Source: Leukemia. 2023 Jul 14;37(9):1767–78. doi: 10.1038/s41375-023-01968-z (PMC10457206; doi:10.1038/s41375-023-01968-z)
Supplement: Supplementary file 1 — Supplementary Table 1 [file 41375_2023_1968_MOESM1_ESM.docx]

**Supplementary Table 1.**

Case Reports for Pediatric BPDCN Published from 2002 to 2022

| **Study** | **Patient Age, Years/Sex** | **Documented Site of Involvement at Presentation** | | | | | **First- Line Therapy** | **Initial Response** | **Time to Relapse, Months** | **Subsequent Treatment** | **HSCT Timing** |
| --- | --- | --- | --- | --- | --- | --- | --- | --- | --- | --- | --- |
|  |  | Skin | PB | BM | CNS | LN |  |  |  |  |  |
| **Falcão et at., 2002^125^** | 18/M | + |  | + |  | + | CHOP | DOD |  |  |  |
|  | 8/M | + |  | + |  | + | ALL | CR |  |  |  |
| **Feuillard et at., 2002^83^** | 6/F |  |  |  |  | + | ALL | CR |  |  | CR1 |
|  | 14/M | + |  |  |  |  | ALL | CR |  |  |  |
| **Anargyrou et at., 2003^126^** | 17/M | - | + | + |  | - | ALL | CR |  |  |  |
| **Karube et at., 2003^89^** | 17/F | + | - |  |  |  | NHL | CR |  |  | CR1 |
| **Rossi et at., 2006^127^** | 8/M | - | + | + |  | - | ALL | CR |  |  |  |
|  | 14/M | - | + | + |  | - | ALL | CR |  |  |  |
|  | 15/M | - |  | + | - | - | ALL | CR |  |  |  |
| **Ruggiero et at., 2006^90^** | 8/M | + | - | + |  | + | ALL | CR |  |  |  |
| **Eguaras et at., 2007^128^** | 6/F | + | + | + |  | + | ALL | PR | - | None |  |
| **Hu et at., 2007^129^** | 0.7/M | + | - | + |  | - | AML | DOD |  |  |  |
| **Pilichowska et at., 2007^91^** | 16/F | + | - |  |  | + | CHOP+IT | DOD |  |  |  |
| **Hama et at., 2009^85^** | 5/M | + | - | + |  | - | AML | CR | 8 | HSCT | Relapse |
| **Reineks et at., 2009^130^** | 9/F | - | + | + | + |  | ALL | CR |  |  |  |
| **Tsagarakis et at., 2010^131, 132^** | 17/M | - |  | + | + | - | ALL | CR |  |  |  |
| **Dalle et at., 2010^133^** | 8/M | - | + | + |  | + | AML | CR | 6 | Ara-C |  |
| **Jegalian et at., 2010^10^** | 4/F | + | - |  |  | - | NHL | CR |  |  |  |
|  | 7/F | + | - | - |  | - | NHL | CR | - | ALL | CR1 |
|  | 7/M | + | - | - |  | + | ALL | CR |  |  |  |
|  | 8/M | + | - | + |  | + | ALL | CR |  |  |  |
|  | 9/M | + | - |  |  | + | NHL | CR | 9 | BFM-ALL>ICE+RT>HSCT | CR2 |
|  | 10/M | - | - | - |  | - | ALL | CR |  |  |  |
|  | 12/F | + | - | + |  | - | ALL | DNOD |  |  |  |
|  | 15/F | + | - | - |  | + | ALL | CR |  |  |  |
| **Lucioni et at., 2011^134^** | 9/M | + | + | + |  | - | ALL | CR |  |  |  |
|  | 19/M | + | - | - |  | - | NHL | CR | 10 | None |  |
| **Hashikawa et at., 2012^135^** | 15/F | - |  | + |  | + | AML | CR |  |  |  |
| **Gambichler et at., 2013^136^** | 15/F | + | - | - |  | - | AML | CR |  |  | CR1 |
| **Sakashita et at., 2013^49^** | 5/F | + | + | + |  |  | ALL | CR |  |  | CR1 |
| **Tokuda et at., 2014^137^** | 0.1/F | - | + | + |  |  | ALL | CR |  |  |  |
| **Vigemyr et at., 2014^138^** | 11/M | + | - | + |  |  | ALL | CR |  |  | CR1 |
| **Zhong et at., 2014^139^** | 10/F | + | - | - | - | + | NHL | CR |  |  |  |
| **Dharmani et at., 2015^140^** | 12/F | + | + | + | + |  | ALL | CR | 15 | None |  |
|  | 17/F | + | - | + |  | + | ALL | CR | 5 | None |  |
| **Liu et at., 2015^141^** | 2/F | + | - | - | - |  | ALL | CR |  |  |  |
| **Nguyen et at., 2015^142^** | 13/F | + |  |  |  |  | ALL | CR |  |  | CR1 |
|  | 15/M | + | - | + | - |  | ALL | CR |  |  |  |
| **Sheng et at., 2015^143^** | 6/F | + |  |  |  |  | None | DOD |  |  |  |
| **Yang et at., 2015^144^** | 8/F |  | + | + |  |  | AML | CR |  |  |  |
| **Martín-Martín et at., 2016^93^** | 11/M | - |  | + | + |  | ALL | CR |  |  |  |
|  | 16/M | + |  | + | + |  | ALL | CR | 5 | None |  |
| **Shimomura et at., 2016^145^** | 9/F | + | - | - | - |  | ALL | CR |  |  | CR1 |
| **Deng et at., 2017^146^** | 7/F | + |  | + |  | + | ALL | PR |  |  |  |
|  | 9/M | + |  | + |  | + | AML | NR | - | ALL | CR1 |
| **Yigit et at., 2017^147^** | 11/F | + | - | - |  |  | ALL | CR |  |  |  |
| **Diness et at., 2018^148^** | 5/F | + |  | + |  |  | ALL | CR |  |  |  |
| **Rajkumari et at., 2018^149^** | 6/F |  | + | + |  | + | ALL | PR | - | HSCT |  |
| **Sumarriva et at., 2018^150^** | 6/M |  | + | + |  | + | ALL | CR | 19 | ALL>HSCT |  |
| **Sun et at., 2018^100^** | 10/F | + |  | + |  |  | ALL | CR | 22 | Decitabine |  |
|  | 12/F | + |  |  |  | + | Tag | PR | - | CVAD>HSCT | CR2 |
|  | 15/F | + |  | + |  |  | ALL | CR | 12 | BDMV |  |
| **Cernan et at., 2020^151^** | 5/M | + |  | + | + | + | AML | CR | 2 | AML (2004) + RT > HSCT | CR1, CR2 |
|  | 8/F | + |  | - |  | - | ALL | CR |  |  |  |
|  | 16/M | - |  | + |  | + | ALL | CR |  | AML BFM (2012) > HSCT | CR2 |
|  | 18/F | - |  | + |  | - | CHOP | PR | - | Alemtuzumab > HSCT > Relapse > DOD | PR |
| **Demiröz et at., 2020^152^** | 11/M | + |  | + |  |  | ALL | CR |  |  |  |
|  |  |  |  |  |  |  |  |  |  |  |  |
| **Chan et at., 2021^153^** | 6/M | + |  |  |  |  | ALL | CR |  |  |  |
|  | 10/M | + |  | + | + | + | ALL | CR |  |  | CR1 |
|  | 11/F | + |  | + | + |  | ALL | CR |  |  |  |
| **Guo et at., 2021^154^** | 15/F | + |  | + |  |  | CHOP | CR | 11 | VDCLP |  |
| **Liao et at., 2021^47^** | 0.8/F | + |  | + | + | - | ALL | CR |  |  |  |
|  | 6/M | + |  | + |  | + | AML | CR | 12 | None | Relapse |
|  | 7.7/M | + |  |  |  |  | ALL | CR |  |  |  |
|  | 11.7/F | + |  |  |  |  | ALL | CR |  |  |  |
| **Rivas-Calderon et at., 2021^155^** | 13/M | + |  | - | - |  | ALL | CR |  |  | CR1 |
| **Tanchiva et at., 2021^156^** | 11/M | + | - | - |  | + | ALL | CR |  |  |  |
| **Tekkeşin et at., 2021^157^** | 4.5/F | + | + | + |  | + | ALL | CR |  |  | CR1 |
| **Abla et at., 2022^78^** | 11/M | + | + | + |  |  | ALL | CR | 11 | CVAD>HSCT | CR1, CR2 |

All blank spaces within the columns for site of involvement at presentation represent a lack of explicit documentation of initial findings. PB: peripheral blood, BM: bone marrow, CNS: central nervous system involvement (either via imaging or positive findings on CSF), LN: lymph node, either identified through PET, biopsy, or both. Treatment- ALL: acute lymphocytic leukemia, AML: acute myelogenous leukemia, NHL: non-Hodgkin's lymphoma, CHOP: cyclophosphamide, doxorubicin, vincristine, and prednisone, CVAD: cyclophosphamide, vincristine, doxorubicin, and dexamethasone, IT: intrathecal therapy, BFM: Berlin-Frankfurt-Münster ALL protocol, BDMV: bortezomib, dexamethasone, mitoxantrone, and vinorelbine, ICE: ifosfamide, carboplatin, etoposide, RT: radiation therapy, CR1: first complete remission, CR2: second complete remission, PR: partial response, NR: no response, DOD: dead of disease, HSCT: hematopoietic stem cell transplant
